# Pharmacological Management of insomnia Associated with Parkinson’s Disease

**DOI:** 10.1192/j.eurpsy.2022.2098

**Published:** 2022-09-01

**Authors:** O. Vasiliu

**Affiliations:** Dr. Carol Davila University Emergency Central Military Hospital, Psychiatry, Bucharest, Romania

**Keywords:** Parkinson disease, atypical antipsychotics, Insomnia

## Abstract

**Introduction:**

Parkinson’s disease (PD) is a progressive neurological disorder that associates multiple psychiatric symptoms and disorders, like depression, neurocognitive impairment, sleeping disorders, etc. Insomnia is frequently detected in this population, with a prevalence of over 50% according to several studies.

**Objectives:**

To present a case series dedicated to the treatment of insomnia in patients diagnosed with PD, who did not meet diagnostic criteria for any other psychiatric disorder.

**Methods:**

A number of three patients (2 male, one female, mean age 65.2 years) diagnosed with PD, were evaluated for insomnia. They were all initiated on quetiapine XR 50 mg QD, and up-titrated according to the individual response. All these patients were undergoing treatment for their neurological disease, which remained stable for the next 3 months. A structured clinical evaluation was performed monthly, and safety measurements were also performed. All patients self-evaluated their insomnia severity on a 10-point visual analogic scale (VAS).

**Results:**

After 3 months, patients reported a favorable evolution of their insomnia- VAS score improved significantly to baseline (from 7.3 to 3.3), without significant adverse events (metabolic parameters and QTc values did not change significantly during the treatment period). Daytime sleepiness was not reported as being significant by any of these patients. The mean dose of quetiapine XR used was 75 mg QD (50-150 mg QD), and the mean duration of the needed treatment for insomnia was 8.3 weeks (4-11 weeks).

**Conclusions:**

Quetiapine XR could be useful in patients with PD-related insomnia, and the mean dose is usually below 100 mg QD.

**Disclosure:**

No significant relationships.

